# Why historians must learn from physiologists

**DOI:** 10.1113/EP093919

**Published:** 2026-06-17

**Authors:** Katherine Hill, Lyndal Roper

**Affiliations:** ^1^ Upland Arts CIC Dumfries, Dumfries UK; ^2^ Faculty of History, University of Oxford Schwarzman Centre, Radcliffe Observatory Quarter, Oxford

We (Hill and Roper) are two historians who became interested during the pandemic in the effect of physical exercise on thinking. We started to use physical exercise before or in the middle or at the end of the classes we taught, one of us at Birkbeck, University of London, with diverse student groups of different ages, and the other at the University of Oxford, with a homogeneous student population albeit with very different levels of fitness. We tried different kinds of physical exercise, some demanding boot camp‐style exercises, in addition to a set produced by The Athletes’ Centre (TAC) Oxford, an independent gym that has designed sets of exercises that take 3 min.

At the same time, we became highly concerned with the growth of artificial intelligence (AI) and the largely hostile attitudes our colleagues had towards its use. We were convinced that AI could also help historians in writing and in asking new questions, and we hoped that the growing use of AI would mean that critical thinking and originality would become even more important skills. If our students are to be able to operate in the world of AI, they need to be able to examine the relationship between evidence and the syntheses that AI produces, and to assess sources critically. Above all, humans are embodied; AI is not. That fact now seems to us not merely philosophical, but physiological. Human thought emerges from one of the most metabolically expensive organs, whose performance depends continuously on circulation, gas exchange, substrate delivery and the maintenance of neurovascular homeostasis (Bailey, 2019a, 2019b). In that sense, critical thinking is not only a cultural achievement, it is a biological event.

We wanted to take this further to explore how exercise affects thinking and creativity. By chance, we happened to hear the late Michael Moseley's Radio 4 programme ‘Just One Thing’ on physical exercise, which drew on Professor Damian Bailey's work (https://www.bbc.co.uk/sounds/play/m000vp09). *One of us became irritated with how our universities did not teach critical or original thinking to History graduate students; either you had it or you didn't, seemed to be the attitude*. But we felt sure that it could be taught. So, we designed sets of simple exercises to improve critical thinking; for instance, getting people to separate out argument from empirical findings and to summarise the findings without reference to the argument, which is surprisingly difficult for people to do. This enables students to interrogate the relationship between argument and evidence, to work out what the historian did, what sources they used, and to ask whether the evidence does support the argument. And it helps students to look at arguments, whether they follow logically, how precise or imprecise their claims are, and what they might be leaving out.

We next developed these workshops using other tasks too, working out sets of intellectual techniques that could be identified and practised, and we incorporated a break in the middle of the workshops for exercise. We piloted them in Oxford with four different groups, in Glasgow with two groups, and in Durham also with two groups, and we called them ‘Moving History’. Figure 1 illustrates one of the ‘Moving History’ workshops in action, showing participants undertaking a brief exercise break intended to perturb sedentary cognitive routine and engage the embodied physiological processes that might support renewed critical and creative thinking.

**FIGURE 1:**
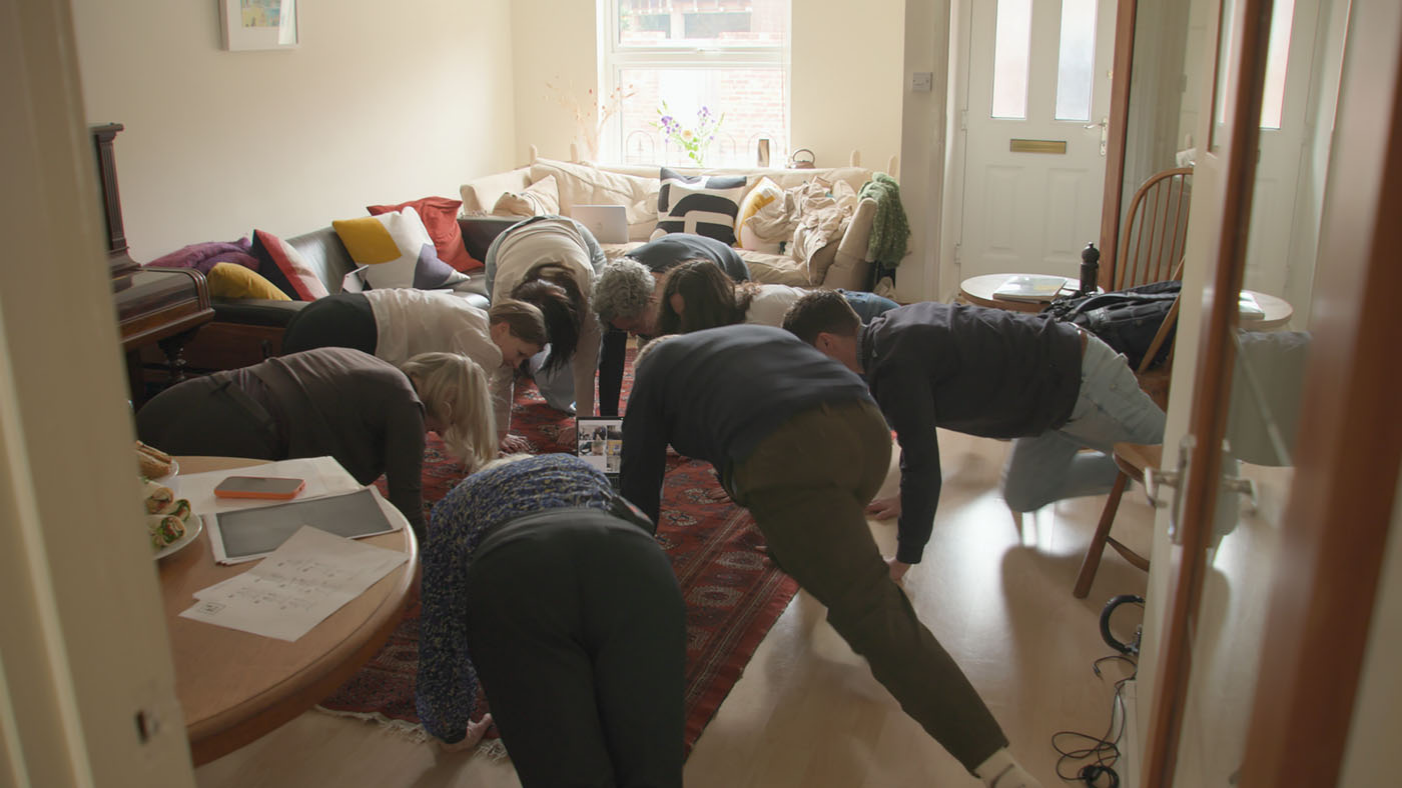
A typical ‘Moving History’ workshop.

By chance, one of us happened to be listening to Radio 4 when the same ‘Just One Thing’ programme was repeated after Michael's death, and this time we listened for the name of the researcher. Damian Bailey replied at once. Sometimes encounters have to find their right time, and this was it. Had we contacted Damian when we first heard the programme, we would not have been ready to develop the workshops, but it just so happens that both of us are able to do so now. We were astonished to realise that the exercises from the Oxford Athletes’ Centre do exactly what Damian says is so important: they last for 3 min, and they involve moving the head in the three postural planes. And yet neither knew of the other's work!

That convergence matters physiologically. A short bout of exercise is capable of altering cerebral haemodynamics instantaneously. The brain has an exceptionally high demand for oxygen and glucose, and even modest changes in cerebral perfusion can alter the rate at which these substrates are delivered to active neural tissue (Bailey, 2019a, 2019b). Exercise does not simply ‘wake people up’; it imposes a controlled cardiovascular stimulus that changes cardiac output, arterial pressure, arterial carbon dioxide tension and cerebrovascular shear, all of which influence cerebral blood flow. Across the lifespan, higher cardiorespiratory fitness is associated with elevated cerebral perfusion and cerebrovascular reactivity, suggesting that habitual exercise might help to preserve the biological machinery that supports cognition (Bailey et al., 2013).

The likely mechanistic pathway is integrative. Repeated increases in cerebral blood flow expose the cerebrovascular endothelium to changing patterns of pressure, strain and shear stress. These mechanical forces are not passive: they are sensed by endothelial structures including the glycocalyx, integrins, ion channels and caveolae, which trigger intracellular signalling cascades linked to nitric oxide bioavailability, angiogenic signalling and vascular adaptation. In Bailey and colleagues’ framework, these haemodynamic changes are accompanied by redox‐sensitive signalling, with reactive oxygen and nitrogen species acting not only as agents of damage when excessive, but also as physiological signals when appropriately constrained, helping to regulate vascular adaptation, trophic factor expression and, ultimately, neuroprotection (Bailey et al., 2018; Calverley et al., 2020).

With Damian's help we devised some tests to see whether movement fostered creativity too. We got the students to write down a thought/idea in relationship to their research on a card before they did some exercise, then immediately after the exercise break. We used different colours (green and pink) to distinguish the ‘before’ from the ‘after’. The next day, in the second workshop, we divided the workshop in half and gave them the pink/green cards the other group had written (this meant that they were not looking at their own ideas or those of others in their group). We asked them to compare them and decide which were better, green or pink; then we teased this out a little by asking which were more abstract, which more creative and which stronger.

The groups responded in interestingly different ways to this task. One group marked the ideas collectively and discovered that the green ideas (‘befores’) scored six to seven whereas the pink scored eight, which they considered to be a marked difference. The other group laid out all the cards and started comparing them and creating a large scale; they had difficulty in reaching a conclusion, took longer, and argued about which ideas they liked. Also interesting was what happened physically at this point. Of their own accord, most of them got off their chairs and sat on the floor; that is, they moved out of their customary academic sitting postures and really worked together, bending towards each other.

We do not claim that exercise straightforwardly ‘causes genius’, nor that cerebral blood flow alone explains originality. The relationship between acute exercise and cognition is more nuanced than that (Tsukomato & Bailey, 2026). Evidence suggests that some components of executive function, particularly inhibition and aspects of attentional speed, might improve during or after exercise, whereas working memory and cognitive flexibility can show mixed responses depending on timing, task and exercise intensity (**Cantelon & Giles,** 2021). Yet that complexity is precisely the point! If historians want to understand how thinking changes in different bodily states, then physiology gives us a language, a mechanism and a set of measurements with which to ask better questions.

We also asked participants how they felt their own thoughts/ideas had been affected by the exercise; here too, their answers were mixed. Some said they felt that exercise had taken them out of their thinking, that they needed time after the exercise; some felt that their first thoughts had been more considered. They all agreed that their thoughts after exercise had been more concrete, less abstract, possibly more original, and that these thoughts were more productive, more directional. We also asked them to draw how exercise made them feel. One group drew a large, happy face with wide eyes and eyelashes to convey the energy they felt; another drew a square with clear lines as the ‘before’, with the ‘after’ as a square of lines of dots. Strikingly, several conveyed how they felt by drawing question marks or coils shooting out of their heads, one with arrows directed down into the head. We noticed how they wanted to convey that their heads felt full of energy and more open to the outside. We have also asked them similar questions in the post‐workshop questionnaire, and it will be interesting to see if they respond differently when they return to a more academic medium.

What struck us was how often participants intuitively described something akin to cerebrovascular opening‐up: fullness, energy, directional force or greater receptivity. Of course, subjective reports are not haemodynamic measurements, but they are not trivial either. They are a form of lived experience, and one that deserves to be brought into conversation with physiology rather than dismissed by it. The point is not that subjective feeling should replace mechanism, but that it might help to identify which bodily states are worth mechanistic interrogation. In that sense, lived experience can become a hypothesis‐generating partner for experimental physiology.

One particularly intriguing avenue concerns the nature of the exercise stimulus itself. Bailey's group, like others, has long used repeated squat–stand manoeuvres, performed at controlled frequencies, to put the brain ‘under pressure’, force a signal out of the biological noise and probe dynamic cerebral autoregulation, i.e., the brain's capacity to buffer rapid changes in blood pressure and stabilise cerebral perfusion (Bailey, Brugniaux et al., 2019; Burma et al., 2024). These manoeuvres impose a deliberate oscillatory, or sinusoidal, haemodynamic challenge, at different frequencies. They are therefore not merely movements but physiological probes. It is tempting to ask whether some of the brief exercise breaks that seem pedagogically effective might also work because they introduce short‐lived oscillations in flow and pressure that stimulate cerebrovascular responsiveness, perhaps enhancing redox‐regulated endothelial signalling and substrate delivery at exactly the time students are being asked to think afresh. That remains to be tested directly, but it is a compelling example of how a classic physiological manoeuvre might illuminate a humanistic question.

This notion of ‘sinusoidal flow’ is especially appealing. Indeed, intermittent high‐intensity work is proposed to create repeated sinusoidal elevations in cerebral blood flow, velocity and shear, a pattern that might be particularly effective in driving, if not indeed optimising endothelial mechanotransduction and adaptive signalling (Calverley et al., 2020). Historians, of all people, should appreciate that pattern matters as much as quantity; not only how much blood reaches the brain, but how it arrives, with what pulsatility, at what frequency, where and in what behavioural conditions. Physiology therefore allows us to move from vague claims that exercise is ‘good for thinking’ and towards much sharper questions about timing, intensity, posture, breathing, cerebrovascular control and the coupling of bodily movement to intellectual work.

The other thing we are trying to do is to involve the unconscious and the peripheral more, because we think this will also enhance creativity. We do this by bringing in different senses and by asking ‘wild’ questions, such as: if this academic essay were a piece of music, what sort would it be? Or, what colour do you associate with this essay? This question, which we originally thought absurd, always yields very interesting results. Sometimes everyone converges on a colour, and the same essay will elicit the same colour from different groups (this made us wonder whether there had been communication between the two groups!). Sometimes the room polarises, and we will find that those reading academic essay A come up with different colours, e.g., red/yellow/orange, but on the same spectrum, whereas those reading essay B come up with black/brown. The colour question allows them to talk about the ‘warmth’ of a writer and to get at other non‐obvious intellectual qualities, and we noticed that they then began to refer to the author by name and to ask about their lives, whereas up to that point no‐one had been able to mention the name of any of the article writers we discussed, generally referring to them as ‘they’.

This, too, might have a physiological dimension. Exercise is not only a vascular event but a multisystem integrated perturbation involving autonomic activation, interoceptive awareness, proprioceptive input and changes in arousal. It might therefore alter not only speed of thought but style of thought, biasing cognition away from static, overlearned pathways and towards more exploratory, creative or associative modes. Whether that translates into better historical interpretation remains an open empirical question. But it is exactly the kind of question that becomes newly tractable when historians, physiologists and other discipline specialists begin to work together. It is a union of multidisciplinary specialists without borders!

As a result, we have chosen to involve a Jungian psychoanalyst, Sophie López‐Welsch, as an advisor on our workshops, so that we are able to develop more ‘left‐field’ questions and think of other methods for attending to the unconscious dimensions in historical source material. We will also continue to think about bringing in varied kinds of sensory inputs (for instance, as an ice‐breaker on the second day we asked people what their favourite food was). We also think it is very important not to do too much, so we are careful not to exceed 3 min of exercise and to offer modified versions of the exercises too. We would never, for instance, ask people about their dreams, because this would be very exposing; we would never ask people to perform a burpee.

That caution is sensible both physiologically and pedagogically, because the dose matters. Exercise that is too intense, too prolonged or too poorly timed might impose competing demands on cognition, particularly on higher‐order executive processes. The emerging literature suggests that moderate or carefully titrated bouts might be more consistently beneficial than indiscriminately hard exercise, especially when the cognitive task follows shortly afterwards rather than coinciding with peak physiological strain (Cantelon & Giles, 2021).

We are really excited about the possibilities of working with physiologists because thinking is embodied. Damian's work has helped us hugely to understand why 3 min of exercise makes such a difference, whether or not the participants are aware of it, whether or not they can put that into words or are consciously aware of all the different dimensions involved. We think it came out in how they moved, in what they drew and in what they said. There is clearly a lot going on here about cerebral blood flow, and that is also what the students drew.

What excites us most is that this collaboration shows the power of physiology in the richest sense (Bailey, 2022). Physiology does not stop at the clinic, the laboratory or the treadmill. It gives us a framework for understanding how movement, circulation, oxygen delivery, glucose availability, autoregulation and neurovascular signalling might shape activities often treated as purely intellectual: judging evidence, revising arguments, making unexpected connections or producing original ideas. The point is not to reduce history to blood flow. It is to recognise that scholarship is performed by living bodies and that the boundaries between humanities and physiology were always more historical than natural.

This also opens a wider conversation about difference. Different students, ages, fitness levels, bodily histories and comfort with movement are likely to experience these interventions differently. It reminds us that both classrooms and laboratories need to attend more carefully to lived experience, inclusion and heterogeneity and that the best future work will almost certainly be collaborative: historians with physiologists, yes, but also with psychologists, educators, therapists and, crucially, the students themselves.

We also think more broadly that it will enrich our account of humans in the past, of human agency and of the relationships of humans to their environments, because it will allow historians to incorporate questions of space and movement, and of both unconscious and conscious motivation, into their work (Roper, 2025; Roper, 2026). After all, it was only in the 18th century that sciences and humanities separated from one another, and now we need to bring them back together!

If originality is a kind of Holy Grail in higher education, then perhaps one route towards it is surprisingly old fashioned: get up, move, perturb the system, restore flow and think again. Physiology has no boundaries. That is precisely why historians must learn from physiologists!

## AUTHOR CONTRIBUTIONS

Lyndal Roper and Katherine Hill conceived the idea and wrote the first draft of the manuscript. Both authors approved the final version submitted for publication and agree to be accountable for all aspects of the work in ensuring that questions related to the accuracy or integrity of any part of the work are appropriately investigated and resolved. Both persons designated as authors qualify for authorship, and all those who qualify for authorship are listed.

## CONFLICT OF INTEREST

None declared.

## FUNDING INFORMATION

None.
